# Adolescent Mental Health and Health-Related Behaviors Across Language-Based School Systems in South Tyrol, Italy

**DOI:** 10.3390/ejihpe16070087

**Published:** 2026-06-25

**Authors:** Christian J. Wiedermann, Verena Barbieri, Giuliano Piccoliori, Doris Hager von Prainsack Strobele

**Affiliations:** Institute of General Practice and Public Health, Claudiana College of Health Professions, 39100 Bolzano, Italy

**Keywords:** adolescent mental health, multilingual school systems, school language, home–school language mismatch, psychosomatic symptoms, sleep behavior, health-related quality of life, digital media use, social support, population-based study

## Abstract

Adolescents growing up in multilingual regions experience diverse educational contexts that may shape their daily routines and psychosocial environments, but their independent relevance for mental health remains unclear. South Tyrol, with its parallel German-, Italian-, and Ladin-language school systems, provides a unique setting to examine these associations. This study assessed whether school language and home–school language mismatch are associated with mental health, psychosomatic symptoms, and health-related behaviors among adolescents. We analyzed data from a population-based survey of 2005 adolescents aged 11–19 years who provided self-reported information on mental health, psychosomatic complaints, school stress, social support, digital behaviors, lifestyle, and sleep. Multivariable regression analyses examined the independent association of home–school language mismatch with mental health outcomes, adjusting for sociodemographic and educational factors and further incorporating sleep-related behaviors. Mental health outcomes, psychosomatic symptoms, and most health-related behaviors showed little variation by school language, with generally small effect sizes. Home–school language mismatch was associated with slightly higher depressive symptom scores in unadjusted analyses but was not independently associated with mental health outcomes after adjustment. In contrast, weekly sleep problems emerged as the strongest correlate of depressive symptoms, accounting for a substantial proportion of explained variance. These findings indicate that adolescent mental health in this multilingual context is associated less with the language of schooling itself than with broader behavioral and developmental factors, highlighting sleep-related behaviors as a central and modifiable target for prevention.

## 1. Introduction

Studies indicate that the language environment in schools and the wider cultural-linguistic context have been considered as possible contextual factors affecting mental health, with varying empirical results across different settings. Proficiency in the language of instruction and culturally inclusive school climates are associated with better psychological adjustment, whereas language barriers, acculturative stress, and experiences of discrimination increase the risk of mental health problems (Cavicchiolo et al., 2020; Schachner et al., 2016, 2021; Shi et al., 2019; Taušová et al., 2019). Supportive teacher practices that foster emotional scaffolding and a sense of belonging appear protective, while students from minoritized home-language backgrounds may report lower perceived support within school settings (Barker et al., 2023; Humeau et al., 2024; Pan et al., 2023), at the same time, findings remain heterogeneous. Linguistic diversity may promote openness and integration in some contexts, whereas linguistic insecurity or mixed effects of cultural and language maintenance have been reported in others, highlighting the importance of resilience, school connectedness, and contextual factors as potential buffers (Hasnain et al., 2024; Khawaja et al., 2017; Piccardi et al., 2022; Q. Yu et al., 2024).

Population-based research from South Tyrol, Italy, provides important context for examining adolescent mental health in a culturally diverse European region. Data from the “Corona and Psyche—South Tyrol” (COP-S) project indicate elevated levels of psychological distress and impaired health-related quality of life among adolescents during and after the COVID-19 pandemic, highlighting mental health as a major public health concern in this population (Barbieri et al., 2022, 2023a, 2023b, 2024a, 2024b). Across survey waves, consistent gender differences have been observed, with girls reporting higher levels of emotional and psychosomatic symptoms (Barbieri et al., 2023b)—a pattern documented across European and international adolescent populations (Patton et al., 2016). These findings underscore the relevance of school- and family-related contexts for adolescent well-being in South Tyrol and motivate further investigation of structural and contextual determinants within the regional school system.

South Tyrol provides a distinctive natural setting for examining contextual determinants of adolescent well-being through its parallel, language-based school systems. Education is organized separately for German-, Italian-, and Ladin-speaking populations, with instruction primarily delivered in students’ first language and largely linguistically segregated school environments (Dekret des Präsidenten der Republik, 1972). While pupils are required to learn the other provincial language and English, everyday schooling remains mostly separated by language, except in Ladin schools, where bilingual instruction is standard. At the same time, families are free to enroll their children in schools outside their language group, resulting in increasing permeability between systems (Schmidt, 2024). In practice, this has led to asymmetric enrollment patterns. While Italian-speaking families increasingly opt for German-language schools, particularly at the elementary level, the reverse pattern is rare. Many of these students return to Italian-language schools at upper secondary level. This selective permeability, combined with a higher concentration of students with migration backgrounds in Italian-language schools, particularly in urban areas, creates distinctive compositional patterns across school types. This combination of institutional separation, selective permeability, and the specific bilingual structure of Ladin schools creates a unique context for studying how language of instruction and home–school language alignment relate to adolescent mental health. Provincial policy promotes multilingual education through initiatives such as Content and Language Integrated Learning (CLIL) and programs enabling temporary enrollment in schools of other language groups to enhance second-language proficiency and intercultural competence (Watschinger, 2019).

This constellation of institutional linguistic separation, increasing permeability through school choice, and explicit policy efforts to promote multilingual competencies and inclusion creates a unique natural laboratory. Within a shared socioeconomic, welfare, and health policy framework, South Tyrol allows the examination of how the language of instruction and associated school climate relate to mental health and psychosomatic symptoms of adolescents, with direct implications for school psychology services, prevention strategies, and media education policies (Cavicchiolo et al., 2020; Piccardi et al., 2022; Schachner et al., 2016, 2021).

This study examined associations between school language context and adolescent mental health across multiple domains. We assessed depressive symptoms (PHQ-2), anxiety symptoms (SCARED-GAD), health-related quality of life (KIDSCREEN-10), and psychosomatic complaints including headaches, abdominal pain, irritability, and sleep problems (HBSC checklist). We also investigated behavioral correlates including sleep quality (weekly sleep problems and usual bedtime), problematic social media use (Bergen Social Media Addiction Scale), and perceived social support (Multidimensional Scale of Perceived Social Support). These measures provided comprehensive assessment of mental health, somatic complaints, and modifiable behavioral factors in relation to linguistic school context.

We situate this study within an ecological systems perspective (Bronfenbrenner, 1979; Bronfenbrenner & Morris, 2007), viewing school language and home–school language mismatch as contextual characteristics within broader sociodemographic, institutional, and behavioral systems. Language-related variables may influence adolescent mental health through multiple pathways. First, linguistic minority stress (Suárez-Orozco et al., 2018) and cultural mismatch (Vedder & Horenczyk, 2006) theories suggest incongruence between home and school language environments may create psychosocial demands and reduce belonging. Second, language-based school systems may indicate structural differences in family resources, migration background, and social integration (Portes & Rumbaut, 2024). Third, within an ecological framework, language variables may interact with proximal behavioral and developmental factors—particularly sleep quality, social support, and digital media use—that directly shape adolescent mental health outcomes.

Sleep represents a biobehavioral process linked to emotional regulation, cognitive functioning, and psychosocial well-being during adolescence (Tarokh et al., 2016). From a developmental perspective, adolescent sleep is characterized by biologically driven phase delay interacting with early school start times, academic demands, and social media use (Inderkum & Tarokh, 2018). These sleep-related factors may act as proximal determinants within the ecological system, potentially mediating or confounding associations between structural characteristics and mental health outcomes.

Against this background, we tested three competing hypotheses:

**H1** **(Institutional differences).** *School language (German, Italian, Ladin) will be associated with differences in mental health outcomes, reflecting underlying sociodemographic and institutional differences between language groups.*

**H2** **(Linguistic mismatch).** *Home–school language mismatch will be independently associated with mental health outcomes after accounting for sociodemographic factors, reflecting potential stress from linguistic incongruence between home and school contexts.*

**H3** **(Proximal behavioral determinants).** *Behavioral factors—particularly sleep quality, but also social support and digital media use—will account for more variance in mental health outcomes than linguistic or institutional characteristics, reflecting their role as more proximal determinants within the ecological system.*

**Figure 1:**
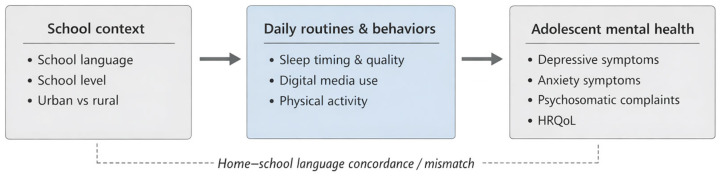
Contextual pathways linking school language and adolescent mental health. School language represents a contextual characteristic that may influence adolescents’ daily routines and behaviors, particularly sleep, which in turn are more strongly associated with mental health and psychosomatic outcomes.

The contextual pathways linking school language and adolescent mental health are shown in Figure 1. If H3 is supported over H1 and H2, it suggests universal, behavior-focused interventions may be more effective than language-specific approaches for promoting adolescent mental health in multilingual educational contexts. However, the cross-sectional design of this study tests associations but does not permit causal inference regarding directional relationships or temporal precedence.

## 2. Methods

### 2.1. Setting, Study Design and Sample

This cross-sectional analysis used data from the fourth wave of the Corona and Psyche—South Tyrol (COP-S) survey series, conducted between 17 March and 13 April 2025. The anonymous online survey targeted families with school-aged children residing in South Tyrol, Italy. Recruitment was supported by public and private school directorates, which distributed personalized survey links via email to approximately 40,000 families.

The survey was administered using the SoSci Survey platform (version 3.2.46; SoSci Survey GmbH, Munich, Germany). It retained the core structure of earlier COP-S waves (Barbieri et al., 2023a, 2024b) including consistent assessment of digital behaviors across all waves, while extending the assessment of post-pandemic psychosocial stressors, school-related factors, and health literacy among adolescents. Wave 4 additionally introduced measures of problematic internet use (PIU) and app-specific use patterns.

For the present study, analyses were restricted to adolescents aged 11–19 years who completed the self-report questionnaire. The survey employed a sequential design: parents first completed a proxy-report questionnaire; adolescents could then access their self-report questionnaire only after parental completion. For each adolescent, one parent independently completed a corresponding proxy-report questionnaire. Adolescent self-reports were used for all mental health, psychosomatic, behavioral, and school-related outcomes, while selected sociodemographic and family characteristics were derived from the parent questionnaire. Participation required written parental consent and adolescent assent. The resulting sample closely approximated the regional age and gender distribution of adolescents according to official provincial statistics. Family language, reported by parents, was used for subgroup and sensitivity analyses to account for South Tyrol’s multilingual context.

### 2.2. Measures

#### 2.2.1. Sociodemographic and Cultural Variables

Adolescents age, gender, school language (German, Italian, Ladin) and school level (elementary, middle, vocational, high/lyceum) were reported by parents. Home language was assessed separately and categorized as German, Italian, Ladin, or Other.

Home–school language mismatch was operationalized as a binary indicator reflecting discordance between the language spoken at home and the language of instruction at school. School language (German, Italian, Ladin) was reported by parents as the official language of instruction attended by the adolescent. Home language was independently reported by parents and categorized as German, Italian, Ladin, or Other. A language mismatch was defined as any case in which the reported home language differed from the reported school language of instruction. Adolescents whose home language and school language were identical were classified as concordant, whereas those with differing languages were classified as mismatched. For the purpose of regression analyses, home–school language mismatch was coded as a binary variable (0 = concordant, 1 = mismatched). For multivariable regression analyses of language mismatch effects, analyses were restricted to adolescents attending German- or Italian-language schools (*n* = 1945). Adolescents attending Ladin-language schools (*n* = 49) were excluded due to small sample size and limited representation in upper secondary education.

This operationalization simplifies the linguistic reality in multilingual South Tyrol, where households may be multilingual, parents code-switch between languages, and adolescents use different languages across contexts. The survey assessed one primary home language and one school language, which does not capture multilingual households, code-switching, or peer language use at school. However, this binary classification provides an initial indicator of language concordance in the two primary domains (home and school) structuring adolescents’ daily experiences.

Among 1984 adolescents with home language data, most reported a single language: German (83.2%), Italian (11.5%), Ladin (2.7%), or Other (2.6%). The observed mismatch rate of 8.0% likely underestimates language complexity, as it reflects only cases where primary home language differed from school language, not multilingual competence or flexible language use.

Place of residence was classified as urban or rural based on official municipal classification according to provincial administrative criteria.

Parental education was reported in the parent questionnaire and classified using the Comparative Analysis of Social Mobility in Industrial Nations (CASMIN) framework into low (primary/lower secondary), medium (upper secondary), and high (tertiary education) (Brauns et al., 2003). Family structure (two-parent vs. single-parent household) and migration background were also parent-reported.

Family socioeconomic status was assessed using the proxy-reported Family Affluence Scale III (FAS III), a validated 6-item instrument covering material assets and family living conditions (Boyce et al., 2006; Currie et al., 2008, 2024). Items were scored according to international guidelines, yielding a total score from 0 to 13. FAS III was analyzed both as a continuous variable and categorized into low, medium, and high affluence based on sample-specific tertiles (Torsheim et al., 2016).

Subjective socioeconomic strain was measured using a single item on perceived financial burden due to rising prices (“perceived inflation”), rated on a 5-point Likert scale from 1 (“not at all burdensome”) to 5 (“extremely burdensome”).

Physical activity was assessed as the number of days per week with at least 60 min of moderate-to-vigorous activity. Sleep characteristics were assessed via reported bedtime, wake time, and subjective sleep quality.

#### 2.2.2. Health Literacy

Adolescent health literacy was assessed using the 10-item Health Literacy for School-Aged Children (HLSAC) instrument (Paakkari et al., 2016). Items assess perceived difficulty in accessing, understanding, appraising, and applying health-related information, with responses rated on a 4-point Likert scale from “very difficult” (1) to “very easy” (4). Total scores range from 10 to 40, with higher scores indicating higher health literacy.

The HLSAC has demonstrated strong psychometric properties in adolescent populations. Validation of the German version using HBSC data confirmed unidimensionality, high internal consistency (Cronbach’s α = 0.88), and measurement invariance (Fischer et al., 2022). The Italian version (HLSAC-I) has shown good reliability and construct validity in representative adolescent samples (Velasco et al., 2021).

#### 2.2.3. Psychological Distress and Psychosomatic Symptoms

Depressive symptoms were assessed using the Patient Health Questionnaire-2 (PHQ-2), a validated 2-item screening tool with items “Little interest or pleasure in doing things” and “Feeling down, depressed, or hopeless” rated on a 4-point scale from 0 (“not at all”) to 3 (“nearly every day”), yielding a total score ranging from 0 to 6. A total score ≥ 3 indicates elevated depressive symptoms (D’Argenio et al., 2013; Kroenke et al., 2003; Schuler et al., 2018).

Anxiety symptoms were assessed using the Generalized Anxiety Disorder subscale of the Screen for Child Anxiety-Related Emotional Disorders (SCARED-GAD). The 9 items include, for example, “I worry about things working out for me” and “I worry about the future” and are rated on a 3-point scale from 0 (“not true”) to 2 (“very or often true”), yielding a total score ranging from 0 to 18; scores ≥ 9 indicate clinically relevant anxiety symptoms (Birmaher et al., 1999; Crocetti et al., 2009; Weitkamp et al., 2010).

Psychosomatic complaints (headache, abdominal pain, irritability, sleep problems) were assessed using the Health Behavior in School-aged Children Symptom Checklist (HBSC-SCL) proxy version (Haugland et al., 2001; Heinz et al., 2022). Responses were dichotomized into weekly or more frequent versus less frequent symptoms.

Health-related quality of life (HRQoL) was assessed using the 10-item KIDSCREEN questionnaire (KIDSCREEN-10), a validated generic measure of well-being in children and adolescents. Items cover physical, psychological, and social dimensions of daily functioning and include, for example, “Have you felt fit and well?” and “Have you been able to pay attention?”. Responses were transformed into internationally standardized T-scores using Rasch modeling (mean = 50, SD = 10), with higher scores indicating better perceived quality of life (Ravens-Sieberer et al., 2014). The KIDSCREEN-10 has demonstrated good reliability, cross-cultural validity, and measurement invariance in European adolescent populations and has been used consistently across COP-S survey waves.

Perceived school stress was measured using a single item (“How stressed do you feel by schoolwork?”) on the subjective burden of school demands, rated on a 5-point Likert scale from 1 (“not at all stressed”) to 5 (“very stressed”).

#### 2.2.4. Family Dynamics

Perceived social support was measured using the Multidimensional Scale of Perceived Social Support (MSPSS), a 12-item scale rated on a 7-point Likert scale (Amati et al., 2007; Boggatz, 2023; Zimet et al., 1988). Family and peer subscales were analyzed separately, with higher scores indicating stronger perceived support.

Perceived parental fairness (“My parents treat me fairly”) and parental involvement in school-related matters were assessed using single self-developed items rated on 5-point Likert scales.

Parents also reported whether digital parental control tools (e.g., Family Link, Apple Family Sharing) were used when the child accessed digital media (yes/no).

#### 2.2.5. Problematic Internet Use

Problematic Internet use (PIU) was assessed using the 15-item Generalized Problematic Internet Use Scale 2 (GPIUS-2), covering five dimensions: preference for online social interaction, mood regulation, cognitive preoccupation, compulsive use, and negative outcomes (Caplan, 2010). The scale has demonstrated excellent reliability and validity in German and Italian versions (Barke et al., 2014; Fioravanti et al., 2013).

Problematic social media use was assessed using the 6-item Bergen Social Media Addiction Scale (BSMAS) (Andreassen et al., 2017). Items are rated on a 5-point Likert scale ranging from 1 (very rarely) to 5 (very often), yielding a total score between 6 and 30, with higher scores indicating greater risk of social media addiction. The BSMAS has demonstrated good psychometric properties and has been validated in German and Italian adolescent samples (Monacis et al., 2017; Montag et al., 2015).

Daily time spent on digital media for educational and non-educational purposes was recorded separately.

#### 2.2.6. Late Bedtime and Poor Sleep Quality

Late bedtime was defined as a usual bedtime after 23:00 on schooldays, derived from adolescents’ self-reported bedtime. Responses indicating bedtime later than 23:00 were coded as late bedtime (yes), and earlier bedtimes as no.

Poor sleep quality was defined as the presence of self-reported sleep problems occurring at least once per week, based on the weekly sleep problems item from the HBSC symptom checklist. Responses indicating symptoms “weekly” or “more than weekly” were coded as poor sleep quality (yes), and less frequent symptoms as no.

### 2.3. Statistical Analysis

A sample of approximately 2000 adolescents provides adequate statistical power to detect small to moderate effect sizes between groups at α = 0.05 across the range of statistical tests employed, as established in conventional power analysis frameworks (J. Cohen, 2013; Faul et al., 2007). No post-stratification or statistical weighting was applied.

Descriptive statistics are presented as means with standard deviations for continuous variables and frequencies with percentages for categorical variables. Group differences by school language (German, Italian, Ladin) were examined using χ^2^ tests for categorical variables and one-way ANOVA or Kruskal–Wallis tests for continuous variables, depending on distributional assumptions (Field, 2024).

Bivariate associations between continuous variables were assessed using Spearman’s rank correlation coefficients. Spearman rank correlations were used because several variables were measured on ordinal scales (e.g., Likert-type responses) and preliminary inspection revealed non-normal distributions in key variables (Field, 2024). Differences in psychological distress, psychosomatic symptoms, health literacy, and digital behaviors across school language groups were tested using χ^2^ tests and ANOVA/Kruskal–Wallis tests as appropriate.

Missing data were assessed using Little’s MCAR test (Little, 1988) implemented in the naniar package (Tierney & Cook, 2023) for R version 4.5.1. The test indicated that data were not missing completely at random (χ^2^ = 17.71, df = 9, *p* = 0.039), primarily due to systematic absence of HRQoL data for a substantial proportion of the sample. Missing data rates varied by measure: PHQ-2 and SCARED-GAD (20%), and health-related quality of life (80%). The higher missing rate for HRQoL was due to incomplete coverage of this measure across the full sample, reflecting variation in survey administration procedures. Given the pattern of missingness and our analytic approach focusing on complete cases for each specific analysis, we used variable-specific listwise deletion. Sample sizes for each analysis are reported in tables and vary by measure.

Multivariate logistic regression models were fitted to identify independent predictors of (i) elevated depressive symptoms (PHQ-2 ≥ 3), (ii) elevated anxiety symptoms (SCARED-GAD ≥ 9), and (iii) frequent psychosomatic complaints (weekly or more) (Hosmer et al., 2013). Covariates included school language, home language, age, gender, migration background, family structure, parental education (CASMIN), family affluence (FAS III), health literacy (HLSAC), perceived school stress, peer and family support (MSPSS), and problematic digital behaviors (GPIUS-2 and BSMAS).

Prior to multivariable modeling, we examined correlations among all predictor variables to identify potential multicollinearity. Pearson correlation coefficients were calculated for continuous predictors, and variance inflation factors (VIFs) were computed in preliminary regression models. School level showed near-perfect correlation with age (r ≈ 1.00), as both variables capture the same developmental stage within the structured South Tyrolean education system and was therefore excluded from multivariable models to avoid redundancy. Age was retained as a continuous predictor, providing greater statistical precision than the categorical school level variable. Urban/rural residence showed substantial association with school language (Cramér’s V = 0.402, *p* < 0.001) and was not included as a covariate in models examining school language effects to avoid confounding. Variables with VIF values ≥ 5 were not included simultaneously in regression models.

Multiple linear regression was used to examine correlates of problematic Internet use (GPIUS-2 total score). Model assumptions were checked using residual diagnostics; multicollinearity was assessed using variance inflation factors (VIF < 2).

Additional analyses focusing on home–school language mismatch were conducted using multivariable regression models. Unadjusted models examined associations between language concordance versus mismatch and mental health outcomes (PHQ-2 score, SCARED-GAD score, HRQoL, elevated depressive symptoms, elevated anxiety symptoms, weekly headaches). Multivariable models were then fitted adjusting for age, gender, and school language (German vs. Italian).

In a final integrative model examining depressive symptoms (PHQ-2 score), sleep-related variables (late bedtime after 23:00 on schooldays and weekly sleep problems) were additionally included to assess whether the association between language mismatch and depressive symptoms was independent of sleep timing and sleep quality.

Ladin-language schools were excluded from mismatch-related regression analyses due to limited subgroup sizes and missing self-reported HRQoL data.

All analyses were performed using IBM SPSS Statistics (version 27.0; IBM Corp., Armonk, NY, USA). Statistical significance was set at *p* < 0.05 (two-tailed). Given the exploratory nature of group comparisons across multiple outcomes, no adjustment for multiple testing was applied.

## 3. Results

### 3.1. Sample Description

Out of the 2005 participants who provided self-reported data, 1994 responses were deemed valid for language analysis. Table 1 provides a summary of the characteristics of adolescents categorized by the language of their school (German, Italian, Ladin). Most participants attended German-language schools, with smaller proportions enrolled in Italian- and Ladin-language schools. Age differed slightly across groups, with adolescents in Italian-language schools being marginally older and those in Ladin-language schools younger; although statistically significant, the effect size was negligible. Gender distribution did not differ meaningfully by school language.

School level varied significantly across groups, with Italian-language schools showing a higher proportion of students in upper secondary or lyceum tracks and Ladin-language schools a higher proportion of middle school students; the associated effect size was small. Home language was strongly associated with school language, showing a large effect size, with high concordance between school and family language in German- and Italian-language schools and greater language differences among students attending Ladin-language schools.

Adolescents in Italian-language schools more frequently reported a migration background and living in single-parent households compared with those in German- or Ladin-language schools; both differences were statistically significant but small in magnitude. Parental education also differed across school language groups, with Italian-language schools including a higher proportion of adolescents with highly educated parents, whereas German-language schools showed a relatively higher share of lower parental education; effect sizes were small.

Health literacy scores differed marginally across groups, with slightly lower scores among adolescents in Italian-language schools, but the effect size was negligible. Family affluence varied modestly by school language, with lower affluence more common in Italian-language schools and higher affluence more frequent in Ladin-language schools. Urban residence showed the most pronounced between-group difference, with adolescents attending Italian-language schools being substantially more likely to live in urban areas, corresponding to a moderate effect size.

Urban residence was strongly associated with Italian-language school attendance (79.4% of students in Italian-language schools attended urban schools compared to 22.8% in German-language schools; Cramér’s V = 0.402, *p* < 0.001), reflecting the geographic concentration of Italian-language schools in urban centers, particularly Bozen/Bolzano.

Overall, school language groups differed across several sociodemographic characteristics. Most differences were small in magnitude, whereas home language concordance and urban–rural residence represented the most substantial between-group contrasts.

### 3.2. Mental Health and Psychosomatic Outcomes

Internal consistency was assessed for all multi-item scales using Cronbach’s alpha. Reliability ranged from acceptable to excellent across measures. The PHQ-2 (α = 0.73) and SCARED-GAD subscale (α = 0.74) demonstrated acceptable internal consistency. The HLSAC showed good internal consistency (α = 0.89). The MSPSS demonstrated excellent reliability for both the total scale (α = 0.98) and all subscales (Family α = 0.97, Friends α = 0.97, Significant Other α = 0.97). The GPIUS-2 total scale showed excellent reliability (α = 0.93), with subscale reliability ranging from acceptable to good (α = 0.77–0.87). The BSMAS demonstrated good internal consistency (α = 0.85). KIDSCREEN-10 uses Rasch-derived T-scores; reliability is documented in validation studies (Ravens-Sieberer et al., 2014).

Table 2 presents mental health and psychosomatic outcomes stratified by school language group. Mean symptom scores for depressive symptoms (PHQ-2) and anxiety (SCARED-GAD) did not differ significantly across school language groups, and the associated effect sizes were negligible. Similarly, the proportions of adolescents exceeding established cut-offs for elevated depressive or anxiety symptoms were comparable among German-, Italian-, and Ladin-language schools.

The prevalence of weekly psychosomatic complaints was largely similar across groups. Weekly headaches differed statistically by school language; however, the magnitude of this difference was small, indicating limited practical relevance. Weekly abdominal pain, irritability, and sleep problems showed no statistically significant differences between school language groups.

Overall, mental health indicators and psychosomatic symptoms showed little variation by school language. Where statistically significant differences were observed, effect sizes were small, suggesting that school language is not a major determinant of adolescent mental health or psychosomatic well-being in this population.

### 3.3. School Stress and Social Support

Adolescents attending Italian-language schools reported significantly higher levels of school-related stress compared with those attending German-language schools, although the effect size was small (Table 3). No meaningful differences were observed between school language groups in overall perceived social support or family support. Peer support was slightly lower among students in Italian-language schools; however, the associated effect size was negligible.

### 3.4. Digital Behaviors and Problematic Internet Use

Digital media use and indicators of problematic Internet use differed only marginally by school language group (Table 4). Mean daily screen time for school-related and private use was comparable between German- and Italian-language schools, with no statistically significant differences and negligible effect sizes. Overall problematic Internet use, as measured by the GPIUS-2 total score and its subscales, showed no meaningful variation between groups. A borderline difference was observed for the preference for online interaction subscale, with slightly higher scores among students attending Italian-language schools, although the effect size was trivial.

Symptoms of social media disorder, assessed by the BSMAS, were modestly lower in Italian-language schools and reached statistical significance; however, the associated effect size was negligible (ηp^2^ = 0.003, below the 0.01 threshold for small effects), indicating no practical significance. The prevalence of parental control tool use did not differ between groups.

Overall, digital behaviors and problematic Internet use patterns appeared largely similar across school language groups, with observed differences being small in magnitude and unlikely to be of substantive significance.

### 3.5. Lifestyle and Sleep

Lifestyle behaviors and sleep characteristics differed modestly by school language group (Table 5). Adolescents attending German-language schools reported a higher frequency of meeting the recommended level of daily physical activity compared with those in Italian-language schools although the effect size was small.

Mean sleep duration was slightly longer among adolescents in German-language schools than among those attending Italian-language schools (8.27 vs. 8.05 h, *p* = 0.006), again with a small effect size. In contrast, late bedtime after 23:00 was substantially more common among adolescents in Italian-language schools (24.8%) than among those in German-language schools (10.3%). This difference was statistically significant and associated with a small-to-moderate effect size (Cramér’s V = 0.139).

No statistically significant difference was observed in the prevalence of poor sleep quality between school language groups.

### 3.6. School–Home Language Mismatch and Mental Health Outcomes

Among adolescents with self-reported data, 158 of 1994 participants (8.0%) reported a mismatch between the language spoken at home and the language of schooling. The prevalence of home–school language mismatch varied by school language group.

Among students attending German-language schools, 117 of 1727 adolescents (6.8%) reported a language mismatch. In contrast, mismatch was more common among students in Italian-language schools, where 24 of 218 adolescents (11.1%) reported a discrepancy between home and school language. The highest relative proportion of mismatch was observed among students attending Ladin-language schools, with 17 of 49 adolescents (34.7%) reporting non-concordance between home and school language.

As described in Methods (Section 2.2.1), Ladin-language schools were excluded from multivariable regression analyses. Among the 53 adolescents with Ladin as their home language, 32 attended Ladin-language schools (60.4% concordant), while 20 attended German-language schools and 1 attended an Italian-language school. Consequently, all 21 adolescents with Ladin home language included in regression analyses were classified as having home–school language mismatch, reflecting the limited availability of Ladin-language schools at upper secondary levels. All further analyses were therefore restricted to adolescents attending German- and Italian-language schools.

**Figure 2:**
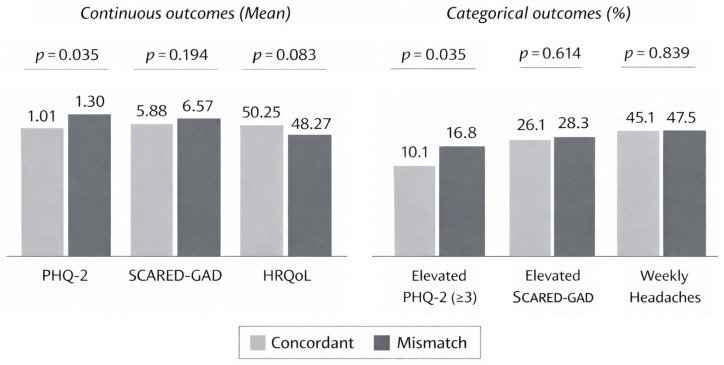
Mental health and psychosomatic outcomes by home–school language concordance. Bars represent mean values for continuous outcomes (PHQ-2, SCARED-GAD, and HRQoL) and percentages for categorical outcomes (elevated PHQ-2, elevated SCARED-GAD, and weekly headaches). Analyses are restricted to adolescents with self-reported data attending German- or Italian-language schools. Group differences were tested using general linear models for continuous outcomes and χ^2^ tests for categorical outcomes. HRQoL is reported as a standardized T-score, with higher values indicating better perceived quality of life. Abbreviations: PHQ-2, Patient Health Questionnaire-2 (depressive symptoms); SCARED-GAD, Generalized Anxiety Disorder subscale of the Screen for Child Anxiety Related Emotional Disorders; HRQoL, health-related quality of life.

Figure 2 summarizes mental health and psychosomatic outcomes among adolescents with concordant versus mismatched school and home language, restricted to German- and Italian-language schools. Analyses were restricted to German- and Italian-language schools.

Adolescents with a language mismatch showed slightly higher mean depressive symptom scores (PHQ-2) and a higher prevalence of elevated depressive symptoms compared with their concordant peers. Although these differences reached statistical significance in unadjusted analyses (*p* = 0.035), the absolute differences were small, and the associations did not persist after multivariable adjustment (Table 6). Anxiety symptoms assessed by the SCARED-GAD scale, health-related quality of life (HRQoL), and the prevalence of weekly headaches did not differ meaningfully between groups, with overlapping distributions and non-significant *p*-values.

To further examine whether the observed differences were independent of sociodemographic and educational factors, multivariable regression analyses were performed (Table 6).

All models were adjusted for age, gender, and school language (German vs. Italian). School language itself was not independently associated with any mental health outcome after adjustment. After adjustment, home–school language mismatch showed a significant association with depressive symptoms (PHQ-2 score, *p* = 0.035) but not with other mental health outcomes.

For continuous outcomes, language mismatch showed a significant association with depressive symptoms (PHQ-2 score: B = 0.27, 95% CI: 0.02–0.52, *p* = 0.035), indicating that adolescents with language mismatch scored 0.27 points higher on the PHQ-2. For anxiety symptoms (SCARED score) and health-related quality of life (HRQoL), confidence intervals included the null value and associations were not statistically significant. In contrast, age and female gender were consistently and strongly associated with higher depressive and anxiety symptom scores, as well as with lower HRQoL.

Similarly, in logistic regression analyses, home–school language mismatch was not significantly associated with elevated depressive symptoms, elevated anxiety symptoms, or weekly headaches after adjustment. Female gender and increasing age emerged as the strongest predictors of elevated symptom burden across all binary outcomes.

For continuous outcomes, explained variance was modest but consistent: PHQ-2 model R^2^ = 0.081 (adjusted R^2^ = 0.078), SCARED model R^2^ = 0.086 (adjusted R^2^ = 0.083), and HRQoL model R^2^ = 0.062 (adjusted R^2^ = 0.059). For logistic regression models, Nagelkerke R^2^ ranged from 0.045 to 0.084, indicating limited but non-negligible explanatory power (elevated PHQ-2: 0.053; elevated SCARED: 0.084; weekly headaches: 0.045). Interaction terms between home–school language mismatch and school language were tested in all models and were not statistically significant, except for weekly headaches, where a nominal interaction was observed; given small subgroup sizes, this finding should be interpreted cautiously.

Overall, these findings indicate that home–school language mismatch shows a modest but significant independent association with depressive symptoms, even after adjustment for age and gender. For other mental health outcomes, observed differences largely reflect underlying age and gender effects rather than language mismatch per se.

Given the prominent differences in sleep-related behaviors observed between school language groups, and the known relevance of sleep for adolescent mental health, an additional integrative model was fitted to examine whether sleep quality accounted for the residual association between home–school language mismatch and depressive symptoms (Table 7). After full adjustment, home–school language mismatch was not independently associated with depressive symptoms (*p* = 0.128). In contrast, weekly sleep problems were the strongest predictor, accounting for the majority of explained variance (partial η^2^ = 0.098). The inclusion of sleep problems increased the explained variance by nine percentage points (ΔR^2^ = 0.092), indicating that sleep quality is a central correlate of adolescent depressive symptoms, whereas language mismatch appears to exert at most a small, non-independent effect.

## 4. Discussion

In this population-based study of adolescents in South Tyrol, mental health, psychosomatic symptoms, and health-related behaviors showed only limited variation by school language. Although adolescents attending German-, Italian-, and Ladin-language schools differed across several sociodemographic and contextual characteristics, school language itself was not independently associated with depressive or anxiety symptoms, health-related quality of life, or most psychosomatic complaints. Small unadjusted differences observed in selected outcomes were largely explained by age, gender, and structural factors such as school level and urban residence. Home–school language mismatch was associated with slightly higher depressive symptom scores in unadjusted analyses; however, this association did not persist after multivariable adjustment. In contrast, sleep-related factors, particularly late bedtime and frequent sleep problems, emerged as the strongest correlates of depressive symptoms, accounting for a substantial proportion of explained variance. Together, these findings suggest that adolescent mental health in this multilingual context is shaped less by the language of schooling per se than by broader developmental, behavioral, and contextual factors.

### 4.1. School Language, Linguistic Context, and Adolescent Mental Health

The present findings are consistent with evidence suggesting that the language of instruction (LOI) is not a strong independent determinant of adolescent mental health once broader sociodemographic, contextual, and behavioral factors are considered. Although linguistic environments are often theorized to be associated with psychosocial well-being through mechanisms such as language proficiency or inclusion, empirical research directly linking LOI to mental health outcomes remains limited and largely indirect.

Existing studies offer only partial analogs. Acculturation research among migrant adolescents shows that host-language proficiency may be associated with lower depressive symptoms and social anxiety; however, these associations are largely mediated by psychological adjustment processes, including self-esteem, perceived acceptance, and social integration, rather than reflecting direct language effects (Shi et al., 2019). When a shared instructional language is widely used, language barriers tend to exert smaller effects on mental health than peer relations and lifestyle factors (Shi et al., 2019).

Broader reviews further support this interpretation. Socioeconomic conditions, school climate, and teacher–student relationships consistently emerge as stronger predictors of both academic and mental health outcomes than linguistic characteristics of schooling (Matar et al., 2024; Rakesh et al., 2025). In this literature, language is primarily conceptualized as a mediator of learning rather than a proximal determinant of emotional well-being. Acculturation frameworks similarly emphasize that peer relationships, identity development, and discrimination experiences are more strongly linked to mental health than language use per se once these factors are accounted for (Shi et al., 2019).

Across these bodies of work, multivariable models indicate that language-related variables show small and often indirect effects on anxiety and depressive symptoms, frequently attenuated after adjustment for social and behavioral factors. This pattern aligns with the present study, in which neither school language nor home–school language mismatch was independently associated with mental health outcomes after adjustment.

From an ecological systems perspective, adolescent mental health is associated with interactions across multiple contextual levels rather than by single institutional characteristics (Bronfenbrenner, 1979; Bronfenbrenner & Morris, 2007). In South Tyrol, parallel school systems operate within a shared welfare, health, and education infrastructure, which may buffer potential psychosocial disadvantages associated with linguistic separation. Consequently, observed differences in urbanicity or migration background should be interpreted as contextual correlates rather than causal effects of school language.

### 4.2. Home–School Language Mismatch

In the present study, home–school language mismatch is more likely a contextual marker or transitional educational choice than a psychosocial risk factor. The absence of independent associations in adjusted models suggests that mismatch should not be conceptualized as harmful per se but interpreted in relation to co-occurring developmental and behavioral factors. The exclusion of Ladin-language schools from mismatch analyses was methodologically justified by small subgroup sizes and the distinct structure of Ladin education.

Direct evidence on home–school language mismatch as an independent determinant of adolescent mental health is limited. No available studies explicitly model language mismatch while simultaneously adjusting for socioeconomic conditions, school context, and behavioral factors. Existing evidence therefore stems from adjacent research on language competence, communication barriers, and contextual determinants of youth mental health.

Research on general language ability indicates that poorer functional language skills are associated with higher levels of depressive symptoms, social withdrawal, and lower self-esteem in adolescents, even after adjustment for family and socioeconomic factors (St Clair et al., 2019). However, these associations appear non-linear and are largely confined to adolescents with markedly low language ability, suggesting a threshold effect rather than a graded risk (St Clair et al., 2019). Similarly, higher-order language difficulties are overrepresented among clinically referred youth and frequently co-occur with broader social-emotional problems (N. J. Cohen et al., 2013). These findings imply that language-related impairment, rather than language difference itself, may be the relevant risk pathway.

Evidence from studies on communication deprivation further supports this distinction. Among deaf adults, inadequate access to a shared language during childhood has been linked to later anxiety, depression, and identity-related distress (McRae et al., 2025). In these contexts, mental health risks appear to arise from persistent communication barriers and social exclusion, rather than from multilingual exposure per se.

Across broader reviews, socioeconomic conditions, family environment, school climate, and social support consistently emerge as stronger predictors of adolescent mental health than language-related variables (Rakesh et al., 2025). When these factors are accounted for, language effects typically attenuate and remain modest (N. J. Cohen et al., 2013; St Clair et al., 2019). The present findings partially align: language mismatch showed a modest but significant association with depressive symptoms (B = 0.27, *p* = 0.035), though considerably smaller than age, gender, and sleep quality (η^2^ = 0.098). The limited scope—significant only for continuous depressive symptoms—suggests language mismatch operates as one factor among many. However, in South Tyrol, asymmetric enrollment patterns may obscure transition-specific effects. The modest association may reflect both genuinely limited magnitude and methodological constraints. Longitudinal research is needed.

However, the binary operationalization does not capture multilingual households, code-switching practices, or peer language use beyond the official language of instruction. A more differentiated understanding would require measures of language proficiency across contexts, peer language practices, and code-switching behaviors, which were unavailable in this dataset.

Furthermore, peer interactions at school may occur in languages other than the official language of instruction. In South Tyrol, code-switching between German, Italian, and Ladin may occur in peer groups. These peer language dynamics were not captured but may play an important role in shaping linguistic identity and social integration, potentially buffering effects of formal home–school language mismatch.

### 4.3. Sleep and Daily Routines

The present findings highlight sleep timing and sleep quality as the most robust and independent correlates of adolescent mental health in this population. Late bedtime and frequent sleep problems showed the largest effect sizes in multivariable models and explained substantially more variance in depressive symptoms than school language, home–school language mismatch, or other structural school-related factors.

Across cohorts, meta-analyses, and longitudinal studies, sleep disturbances are strongly associated with depressive symptoms, anxiety, psychosomatic complaints, and reduced health-related quality of life. Meta-analytic evidence indicates that subjective sleep quality is more strongly related to depressive symptoms than sleep duration alone (O’Callaghan et al., 2021). Prospective studies further demonstrate that sleep problems predict subsequent internalizing and externalizing symptoms even after adjustment for baseline psychopathology, socioeconomic factors, and screen time (Goldstone et al., 2020; Orchard et al., 2020). Objective sleep measures corroborate these findings, with poorer sleep efficiency and greater night-to-night variability forecasting worsening mental health trajectories across adolescence (Thompson et al., 2024).

Late bedtimes, short school-night sleep, and irregular sleep–wake patterns, including social jetlag, have been consistently linked to mood disturbances, anxiety, suicidality, and poorer quality of life (Tarokh et al., 2016; Wang et al., 2021; Yang et al., 2023; Zhang et al., 2017). Importantly, several studies directly comparing sleep with other determinants show that sleep-related variables often retain independent associations with mental health outcomes after controlling for socioeconomic status, school characteristics, and media use, and in some cases explain variance comparable to or greater than single contextual factors (Goldstone et al., 2020; Paulich et al., 2021; Sampasa-Kanyinga et al., 2020).

From a developmental perspective, these findings align with circadian models of adolescence, which emphasize a biologically driven phase delay interacting with early school start times, academic demands, screen exposure, and urban routines (Inderkum & Tarokh, 2018; Tarokh et al., 2016). In this framework, sleep operates as a proximal, behaviorally modifiable pathway through which broader social and environmental pressures are associated with emotional regulation and psychosocial well-being. The present results support this interpretation, suggesting that structural characteristics such as school language may be associated with daily routines only indirectly, whereas sleep timing and quality exert more immediate effects on mental health.

In summary, converging evidence indicates that sleep timing, quality, and regularity are key determinants of adolescent mental health, often mediating or outweighing the influence of distal contextual factors. These findings underscore the importance of sleep-focused prevention strategies, including attention to school schedules, digital media hygiene, and daily routines, as central components of adolescent mental health promotion.

### 4.4. Integration with Previous COP-S Findings

The present findings align closely with the broader body of evidence generated within the COP-S research program and reinforce a coherent interpretation of adolescent mental health determinants in South Tyrol. Across multiple population-based survey waves conducted between 2021 and 2025, COP-S studies consistently documented high and persistent levels of psychosomatic complaints, including headaches, abdominal pain, sleep problems, and irritability, reported by a substantial proportion of adolescents on a weekly basis (Barbieri et al., 2022, 2023b, 2024b, 2025).

Multivariable analyses from these studies repeatedly identified school-related stress, insufficient or irregular sleep, problematic digital media use, and weak peer connectedness as the most robust correlates of depressive symptoms, anxiety, and psychosomatic complaints. In contrast, structural sociodemographic indicators, such as parental education, migration background, or urban–rural residence, showed weaker and less consistent associations once behavioral and relational factors were taken into account (Barbieri et al., 2023b, 2024b). Pronounced gender differences were observed across outcomes, with girls reporting higher symptom burden and greater clustering of internalizing symptoms, a pattern that remained stable across survey waves and analytical models (Barbieri et al., 2025).

Recent analyses from the 2025 COP-S wave further highlighted the role of sleep and daily routines, with late bedtimes and frequent sleep problems emerging as the strongest predictors of depressive symptoms and reduced health-related quality of life, exceeding the explanatory contribution of most sociodemographic variables (Barbieri et al., 2025). Against this background, the largely null associations observed in the present study between school language and mental health outcomes are coherent with prior COP-S evidence. Together, these findings indicate that adolescent mental health in South Tyrol is shaped primarily by developmental stage, gender, behavioral routines, and relational stressors, whereas school language represents a contextual characteristic with limited independent explanatory value.

### 4.5. Strengths and Limitations

This study has several methodological strengths. First, it is based on a large, population-based sample of approximately 2000 adolescents, providing adequate statistical power to detect small to moderate associations and allowing for stratified and multivariable analyses. Second, South Tyrol’s parallel, linguistically organized school systems offer a rare natural experiment to examine the role of language of instruction within a shared socioeconomic, welfare, and health-care context. Third, the study integrates adolescent self-reports with parent-reported sociodemographic information, reducing common-method bias for key structural variables such as school language, school level, parental education, and family affluence.

Several limitations should be acknowledged. The cross-sectional design is a major limitation precluding causal inference. All variables were measured simultaneously, so reverse causality cannot be ruled out. For example, although weekly sleep problems emerged as the strongest correlate of depressive symptoms, the direction of this association cannot be established. Because sleep and mental health were measured simultaneously, depressive symptoms may contribute to sleep difficulties, sleep difficulties may contribute to depressive symptoms, or both may reflect shared underlying psychosocial processes. This ambiguity limits interpretation of sleep as a modifiable intervention target, as the cross-sectional design cannot establish whether sleep improvement would causally improve mental health. Longitudinal studies are needed to establish temporal precedence. Although the overall sample was large, the Ladin subgroup was small (*n* = 49, representing 2% of the sample compared to German 87% and Italian 11%), reflecting the demographic reality of South Tyrol’s school system but providing limited statistical power for detecting small effects. Moreover, the proportion of Ladin-speaking adolescents attending Ladin-language schools in our sample (60%) exceeded population-based estimates (approximately 35%), suggesting selection bias in recruitment or participation. In addition, Ladin schools are predominantly located in rural areas and offer very limited high school provision, resulting in a younger age distribution and restricted variability in school level among Ladin-language students. This limits the comparability of Ladin students with those attending German- or Italian-language schools and justified restricting several multivariable analyses to the latter groups, which remained adequately powered (German *n* = 1720; Italian *n* = 214). Furthermore, health-related quality of life data were missing for a substantial proportion of Ladin-language students, further constraining subgroup analyses for this outcome.

Most outcomes were based on self-reported measures, which may be affected by reporting bias, social desirability, or differential interpretation of items across language groups. While validated instruments were used for mental health outcomes (PHQ-2, SCARED, KIDSCREEN-10), several constructs relied on single-item, self-developed measures (school stress, parental fairness, parental involvement, and psychosomatic complaints). Such measures, while pragmatic for large-scale surveys, have inherent limitations in reliability and construct validity compared to multi-item scales and should be interpreted as global indicators rather than comprehensive assessments.

An additional consideration concerns language mismatch interpretation. Mismatch prevalence was higher in Italian-language schools (11.1%) than German-language schools (6.8%), reflecting migration patterns and selective enrollment. While regression models adjusted for school language, mismatch remained confounded with migration background and urban residence. The modest association between mismatch and depressive symptoms which became non-significant after adjusting for sleep should be interpreted cautiously, as the differential distribution of mismatch across school types may have limited our ability to isolate mismatch effects from correlated demographic factors.

The binary classification of home and school language does not capture the complexity of language use in multilingual South Tyrol. Many households are multilingual with frequent code-switching, and peer language use at school may differ from the official language of instruction. The survey assessed only one primary language per domain, which does not reflect multilingual competence or code-switching practices. Future studies should include measures of language proficiency across contexts, peer language practices, and code-switching behaviors. Additionally, psychosomatic complaints were assessed using single items from the HBSC symptom checklist rather than validated multi-item scales, and no adjustment for multiple testing was applied in exploratory group comparisons across outcomes, which increases the risk of Type I error. Finally, while the study included perceived school stress and social support, it did not incorporate direct classroom- or school-level measures of school climate, such as teacher–student relationships, pedagogical practices, or institutional inclusion policies. Future longitudinal studies incorporating multilevel school climate indicators and objective behavioral measures would be valuable to further disentangle contextual mechanisms linking schooling environments to adolescent mental health.

### 4.6. Implications for Policy, Practice, and Research

At the policy level, sleep and daily routines should be treated as central public health targets for adolescent mental health promotion. Population-based and systems research consistently identifies sleep timing, quality, and regularity as modifiable upstream determinants of depressive symptoms and psychosomatic complaints, shaped by school schedules, academic demands, and digital communication norms (Heemskerk et al., 2024; Sampasa-Kanyinga et al., 2020; Tarokh et al., 2016). Policies addressing school start times, evening workload, and expectations of digital availability may therefore yield broader mental health benefits than interventions focused on single institutional characteristics.

At the same time, the results caution against over-interpreting language-based school structures as primary drivers of mental health inequalities. Preventive psychiatry reviews and global evidence maps indicate that adolescent mental health is more strongly patterned by socioeconomic conditions, school climate, and relational supports than by individual organizational features such as language of instruction (Fusar-Poli et al., 2021; R. Yu et al., 2023). Accordingly, universal school-based mental health promotion should be prioritized over language-targeted approaches.

For schools and school psychology services, the findings support a focus on universal, routine-oriented, and psychosocial interventions, including social–emotional learning, mental health literacy, stress reduction, and lifestyle-focused programs. Such approaches have shown modest but consistent benefits across educational contexts and are more scalable than narrowly targeted interventions (Gee et al., 2021; Ma et al., 2023; Qu et al., 2024). Embedding sleep hygiene and digital habits into school health promotion appears particularly relevant, given their strong links to emotional well-being (Alonzo et al., 2021; Peuters et al., 2024).

Future research should prioritize longitudinal and multilevel designs examining interactions between sleep, digital behavior, and school demands, and should evaluate multicomponent, universal interventions that integrate daily routines with psychosocial support and school-climate change (Fusar-Poli et al., 2021; Heemskerk et al., 2024; R. Yu et al., 2023).

Overall, effective adolescent mental health strategies should emphasize sleep, daily routines, and psychosocial support within universal school-based frameworks, while treating school language as a contextual rather than primary determinant of mental health outcomes.

## 5. Conclusions

In this population-based study of adolescents in South Tyrol, mental health, psychosomatic symptoms, and health-related behaviors showed little systematic variation by the language of instruction. Although school language groups differed in several sociodemographic and contextual characteristics, school language itself was not an independent determinant of depressive or anxiety symptoms, health-related quality of life, or most psychosomatic complaints once age, gender, and structural factors were considered. Similarly, home–school language mismatch was not associated with adverse mental health outcomes after adjustment.

In contrast, behavioral and developmental factors, particularly sleep quality, emerged as the most consistent and substantive correlates of depressive symptoms, explaining substantially more variance than linguistic or institutional characteristics. These findings underscore that adolescent mental health in this multilingual context is shaped primarily by daily routines, psychosocial stressors, and developmental processes rather than by the language of schooling per se.

Overall, the results suggest that multilingual and linguistically parallel education systems can coexist with broadly comparable psychosocial outcomes if adolescents’ behavioral routines, relational environments, and mental health needs are adequately supported. Efforts to promote adolescent well-being should therefore prioritize universal, routine-oriented, and psychosocial interventions over language-specific approaches.

## Figures and Tables

**Table 1:** Sample characteristics of adolescents by school language group (German, Italian, Ladin).

Characteristic	Total	German School	Italian School	Ladin School	*p*-Value	Effect Size
School language, *n* (% of all self-responders, *n* = 1994)		1727 (86.6)	218 (10.9)	49 (2.5)	—	—
Age, mean (SD)	14.39 (2.34)	14.36 (2.37)	14.71 (2.12)	13.96 (2.13)	0.049	0.003 ^1^
Gender (female), *n* (%)	975 (48.9)	837 (48.5)	108 (49.5)	30 (61.2)	0.208	0.040 ^2^
School level, *n* (%)					<0.001	0.086 ^2^
Middle	811 (43.2)	708 (43.7)	76 (35.8)	27 (60.0)		
Vocational	159 (8.5)	152 (9.4)	5 (2.4)	2 (4.4)		
High/lyceum	906 (48.3)	759 (46.9)	131 (61.8)	16 (35.6)		
Home language, *n* (%)					<0.001	0.697 ^2^
German	1624 (81.9)	1601 (93.2)	12 (5.5)	11 (22.4)		
Italian	283 (14.3)	84 (4.9)	193 (88.9)	6 (12.2)		
Ladin	53 (2.7)	20 (1.2)	1 (0.5)	32 (65.3)		
Other	24 (1.2)	13 (0.8)	11 (5.1)	0 (0.0)		
Migration background (yes), *n* (%)	110 (5.6)	82 (4.8)	26 (12.1)	2 (4.1)	<0.001	0.099 ^2^
Family structure: single parent (yes), *n* (%)	243 (12.3)	194 (11.3)	42 (19.3)	7 (14.3)	0.003	0.076 ^2^
Parental education (CASMIN), *n* (%)					0.004	0.063 ^2^
Low	361 (18.3)	331 (19.4)	25 (11.6)	5 (10.4)		
Medium	829 (42.0)	713 (41.7)	88 (40.9)	28 (58.3)		
High	782 (39.7)	665 (38.9)	102 (47.4)	15 (31.3)		
Adolescent Health Literacy (HLSAC) score, mean (SD)	31.51 (5.14)	31.63 (5.15)	30.62 (5.24)	31.28 (4.07)	0.076	0.004 ^2^
Family Affluence (FAS III), *n* (%)					0.190	0.039 ^1^
Low	338 (17.1)	283 (16.6)	49 (22.6)	6 (12.8)		
Medium	1115 (56.5)	976 (57.1)	113 (52.1)	26 (55.3)		
High	520 (26.4)	450 (26.3)	55 (25.3)	15 (31.9)		
Urban residence (urban), *n* (%)	567 (28.4)	393 (22.8)	173 (79.4)	1 (2.0)	<0.001	0.402 ^2^

Percentages are column percentages (within school-language group), except the first row (“School language”), which is % of all self-responders. Missingness varies by variable (valid n): school language, age, gender, and urban *n* = 1994; school level *n* = 1876 (reported descriptively only; not used as covariate due to perfect collinearity with age); home language *n* = 1984; migration background *n* = 1959; single parent *n* = 1983; parental education *n* = 1972; HLSAC *n* = 1384; FAS III (categorical) *n* = 1973. Group differences were tested using χ^2^ tests for categorical variables and general linear models (ANOVA) for continuous variables. Effect sizes are reported as ^1^ partial eta squared (ηp^2^) for ANOVA and ^2^ Cramér’s V for χ^2^ tests. Cramér’s V interpretation: small (0.10–<0.30), moderate (0.30–<0.50), large (≥0.50). Partial η^2^ interpretation: small (0.01–<0.06), moderate (0.06–<0.14), large (≥0.14). Abbreviations: SD, standard deviation; CASMIN, Comparative Analysis of Social Mobility in Industrial Nations (parental education classification); HLSAC, Adolescent Health Literacy Scale; FAS III, Family Affluence Scale III.

**Table 2:** Mental health and psychosomatic outcomes by school language group.

Outcome	Total	German School	Italian School	Ladin School	*p*-Value	Effect Size
PHQ-2 score, mean (SD) Valid *n*	1.0 (1.25) 2005	1.0 (1.24) 1727	1.10 (1.29) 218	0.88 (1.09) 49	0.612	0.001 ^1^
Elevated PHQ-2 (≥3), % Valid *n*	8.7 2005	8.5 1727	9.6 218	6.1 49	0.728	0.018 ^2^
SCARED-GAD score, mean (SD) Valid *n*	3.5 (3.32) 2005	3.6 (3.34) 1727	3.6 (3.29) 218	3.1 (2.95) 49	0.693	0.001 ^1^
HRQoL score, mean (SD) Valid *n*	50.0 (10.10) 1398	50.19 (10.13) 1245	48.77 (9.81) 151	— 0	0.102	0.002 ^1^
Weekly headaches, % Valid *n*	23.6 1994	23.0 1720	27.1 214	24.5 49	0.014	0.077 ^2^
Weekly abdominal pain, % Valid *n*	18.4 1994	18.5 1720	18.2 214	16.3 49	0.980	0.005 ^2^
Weekly irritability, % Valid *n*	32.7 1994	32.5 1720	34.4 214	30.6 49	0.695	0.022 ^2^
Weekly sleep problems, % Valid *n*	27.9 1994	27.6 1720	30.1 214	26.5 49	0.372	0.037 ^2^

Values are reported as mean (SD) for continuous variables and percentages for categorical variables. Percentages are column percentages (within school-language group). Group differences were tested using: General linear models (ANOVA) for continuous outcomes, with partial eta squared (ηp^2^), χ^2^ tests for categorical outcomes, with Cramér’s V. Missing data were handled by variable-specific exclusion. Effect size interpretation: ^1^ ηp^2^: small (0.01–<0.06), moderate (0.06–<0.14), large (≥0.14); ^2^ Cramér’s V: small (0.10–<0.30), moderate (0.30–<0.50), large (≥0.50). Abbreviations: PHQ-2, Patient Health Questionnaire-2 (depressive symptoms); SCARED-GAD, Generalized Anxiety Disorder subscale of the Screen for Child Anxiety Related Emotional Disorders; HRQoL, Health-related quality of life.

**Table 3:** School stress and social support by school language group.

Variable	Total	German School	Italian School	*p*-Value	Effect Size
School stress, mean (SD)	0.41 (0.49)	0.38 (0.49)	0.59 (0.49)	<0.001	0.019
MSPSS total score, mean (SD)	5.81 (1.45)	5.82 (1.48)	5.76 (1.21)	0.579	<0.001
MSPSS family subscale, mean (SD)	5.97 (1.54)	5.96 (1.56)	6.04 (1.31)	0.528	<0.001
MSPSS peer subscale, mean (SD)	5.56 (1.60)	5.59 (1.59)	5.31 (1.60)	0.032	0.003

Analyses are based on adolescent self-reports and restricted to German- and Italian-language schools due to insufficient valid data from Ladin-language students for school stress and social support measures. Group differences were tested using general linear models (ANOVA); effect sizes are reported as partial eta squared (ηp^2^). Effect size interpretation: small (0.01–< 0.06), moderate (0.06–<0.14), large (≥0.14). Valid cases: School stress 1498 (German 1333, Italian 165); MSPSS total 1472 (German 1306, Italian 166); MSPSS family 1489 (German 1322, Italian 167); MSPSS friends 1493 (German 1326, Italian 167). Abbreviations: MSPSS, Multidimensional Scale of Perceived Social Support; SD, standard deviation; ηp^2^, partial eta squared.

**Table 4:** Digital behaviors and problematic Internet use by school language group.

Variable	Total	German	Italian	*p*-Value	Effect Size
Daily screen time (school use), hours, mean (SD)	1.30 (1.21)	1.29 (1.23)	1.41 (1.06)	0.275	0.001 ^1^
Daily screen time (private use), hours, mean (SD)	2.39 (1.28)	2.38 (1.27)	2.48 (1.33)	0.356	0.001 ^1^
GPIUS-2 total score, mean (SD)	38.98 (19.22)	39.00 (19.32)	38.80 (18.51)	0.904	0.001 ^1^
Preference for online interaction, mean (SD)	6.51 (4.45)	6.43 (4.40)	7.19 (4.82)	0.051	0.003 ^1^
Mood regulation, mean (SD)	9.43 (5.53)	9.41 (5.56)	9.58 (5.26)	0.728	<0.001 ^1^
Cognitive preoccupation, mean (SD)	7.18 (4.56)	7.22 (4.59)	6.85 (4.35)	0.358	0.001 ^1^
Compulsive Internet use, mean (SD)	9.70 (5.14)	9.74 (5.18)	9.43 (4.82)	0.502	0.001 ^1^
Negative outcomes, mean (SD)	6.16 (3.82)	6.21 (3.86)	5.74 (3.51)	0.168	0.001 ^1^
Social media disorder (BSMAS score), mean (SD)	12.01 (4.59)	12.10 (4.59)	11.26 (4.55)	0.038	0.003 ^1^
Parental control tools (yes), %	55.7	55.5	57.4	0.628	0.012 ^2^

Values are reported as mean (SD) for continuous variables and column percentages for categorical variables. School language refers to the language of instruction (German vs Italian). Ladin schools excluded due to insufficient valid data. Valid sample sizes: screen time variables *n* = 1333 (German), *n* = 165 (Italian); GPIUS-2 variables *n* = 1306 (German), *n* = 166 (Italian); BSMAS *n* = 1326 (German), *n* = 167 (Italian); parental control *n* = 1322 (German), *n* = 167 (Italian). Group differences were tested using general linear models (ANOVA) for continuous outcomes, with partial eta squared (ηp^2^) as effect size, and χ^2^ tests for categorical outcomes, with Cramér’s V. Effect size interpretation: ^1^ ηp^2^ small (0.01–<0.06), moderate (0.06–<0.14), large (≥0.14); ^2^ Cramér’s V small (0.10–<0.30), moderate (0.30–<0.50), large (≥0.50). Abbreviations: GPIUS-2, Generalized Problematic Internet Use Scale-2; BSMAS, Bergen Social Media Addiction Scale; SD, standard deviation.

**Table 5:** Lifestyle behaviors and sleep characteristics by school language group.

Variable	Total	German	Italian	*p*-Value	Effect Size
Physical activity ≥ 60 min/day, mean days per week (SD)	0.68 (0.47)	0.70 (0.46)	0.58 (0.50)	0.002	0.007 ^1^
Sleep duration, mean hours (SD)	8.24 (0.93)	8.27 (0.93)	8.05 (0.95)	0.006	0.005 ^1^
Late bedtime (>23:00), %	11.9	10.3	24.8	<0.001	0.139 ^2^
Poor sleep quality, %	42.0	42.5	37.7	0.253	0.030 ^2^

Values are means (SD) for continuous variables and percentages for categorical variables. Percentages are column percentages within school language group. Poor sleep quality was defined as the presence of self-reported sleep problems occurring at least once per week, based on the binary indicator derived from the weekly sleep problems item. Group differences for continuous variables were tested using general linear models (ANOVA) with partial eta squared (ηp^2^) reported as effect size. Group differences for categorical variables were tested using χ^2^ tests, with Cramér’s V reported as effect size. ^1^ Partial eta squared (ηp^2^): small (0.01–<0.06), moderate (0.06–<0.14), large (≥0.14). ^2^ Cramér’s V: small (0.10–<0.30), moderate (0.30–<0.50), large (≥0.50). Valid sample sizes: physical activity 1487, sleep duration 1391, late bedtime 1413, and sleep quality 1411.

**Table 6:** Multivariable regression analyses of mental health outcomes by home–school language mismatch among adolescents attending German- and Italian-language schools.

Predictor	B or OR (95% CI)	*p*-Value	B or OR (95% CI)	*p*-Value	B or OR (95% CI)	*p*-Value
	PHQ-2 score		SCARED-GAD score		HRQoL	
Home–school language mismatch	0.27 (0.02; 0.52)	0.035	0.65 (−0.26; 1.56)	0.163	−1.55 (−3.59; 0.48)	0.135
Age (years)	0.14 (0.11; 0.16)	<0.001	0.33 (0.23; 0.44)	<0.001	−0.97 (−1.20; −0.75)	<0.001
Female gender	0.30 (0.17; 0.43)	<0.001	2.22 (1.74; 2.69)	<0.001	−1.90 (−2.93; −0.87)	<0.001
	Elevated PHQ-2 (≥3)		Elevated SCARED-GAD		Weekly headaches	
Home–school language mismatch	1.67 (0.95; 2.93)	0.074	1.12 (0.71; 1.78)	0.623	1.21 (0.65; 2.24)	0.550
Age (years)	1.23 (1.14; 1.32)	<0.001	1.15 (1.09; 1.22)	<0.001	1.16 (1.08; 1.25)	<0.001
Female gender	1.38 (0.97; 1.95)	0.071	2.50 (1.95; 3.21)	<0.001	1.96 (1.38; 2.79)	<0.001

Values are unstandardized regression coefficients (B) with 95% confidence intervals (CI) for continuous outcomes (PHQ-2 score, SCARED-GAD score, HRQoL) derived from general linear models, and odds ratios (OR) with 95% CI for binary outcomes (elevated PHQ-2, elevated SCARED-GAD, weekly headaches) derived from logistic regression models. Reference categories were concordant home–school language, and male gender. Abbreviations: PHQ-2, Patient Health Questionnaire-2 (depressive symptoms); SCARED-GAD, Generalized Anxiety Disorder subscale of the Screen for Child Anxiety Related Emotional Disorders; HRQoL, health-related quality of life (standardized T-score, mean 50, SD 10; higher scores indicate better quality of life).

**Table 7:** Integrative multivariable regression of depressive symptoms (PHQ-2 score), including sleep-related covariates.

Predictor	B	SE	95% CI	β	*p*-Value	Effect Size
Intercept	0.11	0.26	−0.40 to 0.62	—	0.679	—
Home–school language mismatch (yes vs. no)	0.19	0.12	−0.05 to 0.43	0.04	0.128	0.002
Age (years)	0.13	0.01	0.10 to 0.16	0.23	<0.001	0.062
Female gender (vs. male)	0.26	0.06	0.14 to 0.39	0.10	<0.001	0.013
School language (Italian vs. German)	0.77	0.06	0.65 to 0.90	0.30	<0.001	0.098
Weekly sleep problems (≥weekly)	0.20	0.10	0.00 to 0.41	0.05	0.046	0.003

PHQ-2, Patient Health Questionnaire-2 (range 0–6, higher scores indicate more depressive symptoms). Home–school language mismatch indicates discordance between home and school language. Weekly sleep problems: self-reported sleep difficulties ≥ once per week. Models restricted to German- or Italian-language schools; adjusted for age, gender, and school language. Effect size: partial eta squared (ηp^2^); small (0.01–<0.06), moderate (0.06–<0.14), large (≥0.14). Model fit: R^2^ = 0.173, adjusted R^2^ = 0.170, F(5, 1349) = 56.59, *p* < 0.001. Incremental variance from sleep problems: ΔR^2^ = 0.092 (vs. base model R^2^ = 0.081). Collinearity diagnostics: all VIF ≤ 1.01; tolerance > 0.99. Abbreviations: B, unstandardized coefficient; SE, standard error; CI, confidence interval; β, standardized coefficient; ηp^2^, partial eta squared.

## Data Availability

The data presented in this study are available from the corresponding author upon reasonable requests.

## Abbreviations

The following abbreviations are used in this manuscript:

ANOVA	Analysis of Variance
CASMIN	Comparative Analysis of Social Mobility in Industrial Nations
CI	Confidence Interval
CLIL	Content and Language Integrated Learning
COP-S	Corona and Psyche South Tyrol
COVID-19	Corona Virus Disease 2019
DSM-5	Diagnostic and Statistical Manual of Mental Disorders 5
FAS III	Family Affluence Scale III
GAD	Generalized Anxiety Disorders
GPIUS-2	Generalized Problematic Internet Use Scale 2
HBSC	Health Behavior in School-Aged Children
HLSAC	Health Literacy for School-Aged Children
ICD-10	International Classification of Diseases 10
MSPSS	Multidimensional Scale of Perceived Social Support
PHQ-2	Patient Health Questionnaire 2
PIU	Problematic Internet Use
SCARED	Screen for Child Anxiety Related Emotional Disorders
SD	Standard Deviation
SOMEDIS	Social Media Disorder Scale
VIF	Variance Inflation Factors
